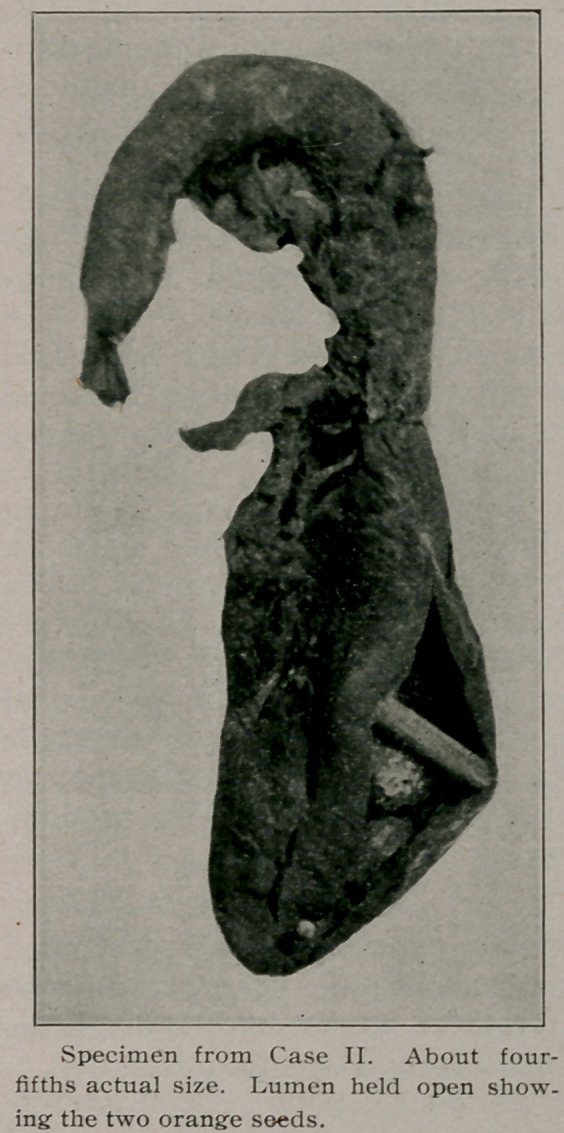# Perilous Calms of Appendicitis1Read at the Surgical Section of the Mississippi Valley Medical Society, Cincinnati, October, 1904.

**Published:** 1905-04

**Authors:** Robert Wallace Hardon

**Affiliations:** Chicago, Ill.


### SELECTIONS.

### Perilous Calms of Appendicitis.[1]

By ROBERT WALLACE HARDON, M. D., Chicago, Ill.

[*Boston Medical and Surgical Journal*, February 16, 1905.]

THE patient died, who only a few hours before seemed to be doing so well, with a more normal pulse and temperature, with practically no pain, able to move about with comparative ease, and when those about him were led to think he was getting well. The surgeon who saw the case, before death, either refused to operate, finding a practically comatose patient, or operated only to find the results of a perforated appendix or the results of bacterial extension, affecting more or less and to a greater or less extent the abdominal contents, and the general system.

Deaver, in the *Journal,* 1904, page 860, says: "Every physician has had one case of severe acute appendicitis, which may have caused difficulty in diagnosis, has referred the patient to a surgeon, stood beside the operating table and observed a highly-

1. Read at the Surgical Section of the Mississippi Valley Medical Society, Cincinnati, October, 1904.

inflamed appendix, perhaps gangrenous, removed. And yet this same physician a few months or years later is called to see another patient in the throes of appendiceal colic, knows well what the disease may lead to, and yet gazes, fascinated as if by a rattlesnake, temporises and dallies until the right iliac fossa becomes ripe and filled with pus."

Why was not the patient operated upon before, at a time when operative results by skilful operators show practically no deaths? First, because of the large percentage of recoveries without operation. Second, because the attending physician or the family or patient hope that this case will be one of a large majority of recoveries without operation. Third, after waiting some hours, or a few days, the patient is apparently improving, and recovery is taking place, with apparently more or less subsidence of the active inflammatory process. This apparent subsidence of symptoms, the more normal pulse and temperature, the lessened pain may be, and frequently is, the precursor of symptoms and conditions much more grave and menacing to life than the more active ones. It often is the treacherous calm to be followed by the death storm, if prompt action is not taken or allowed.

A few examples will serve to illustrate the subject before going further:

Case I.—F. H., 44 years; male; saloonkeeper. Seen November 25, 1903, at noon. A large consumer of whiskey, but never drunk, using about a quart of whiskey daily for many years. Patient obese. Temperature 100° F.; pulse, 96. Arteries hard. Examination of abdomen revealed some pain on pressure, covering practically the entire region below umbilicus on right side. This area was also somewhat dull on percussion.

Ten days previous there was general abdominal pain, supposed to be neuralgic in character. For this an alkaline cathartic was given. Two days following the beginning of the attack, the pain became localised in the right inguinal region, *but was much less intense than at first, and remained in this region up to the time when I first saw the patient.*

No history of chills could be elicited. He was told that he had appendicitis, which had extended to the abdominal cavity, causing peritonitis with pus, and advised to go to the hospital at once and submit to operation. Refusing, he was advised to lie perfectly quiet, given a low enema, and all food and fluid by mouth cut off. Two days later he consented to go to the hospital, the treatment having given him no relief. On entrance at 5 p. m., his pulse was 96; temperature, 101.8°; respiration, 28. Operation was refused at that time. He was kept quiet and given enemas of normal salt solution, one pint every four hours, if awake, and nothing was allowed by mouth. The urine showed ¼ per cent. of albumin, mixed hyaline and granular casts. The blood showed a marked leucocytosis, 29,000 being reported.

The second day in the hospital he had a chill at 2 a.m.; temperature of 104.4°; pulse, 128. Temperature at 8 a.m. next day was 100.6°; pulse, 100. At noon, temperature, 99.4°; pulse, 104. At 8 p. m., temperature, 100.4°; pulse, 90. The following day, after having been seen by two of my colleagues at the hospital he was operated upon, it being considered his only chance. He died. The operation revealed a general peritonitis, with some slight adhesions in the right lower abdomen, holding over a quart of thick, foul pus, having a fecal odor; a sloughed appendix, with only the stump remaining. The treacherous calm had passed; the storm was on, and the wreck came.

Case II.—Miss D. S., aged 26. This patient had been under my care at times for some years. Her past history as concerns this attack was of pain two years ago in the right lower abdominal region, thought to be ovarian, and for which no medical advice was sought.

On March 26, 1904, she ate some crab salad at a restaurant. The next day she had some discomfort in the right iliac region, and burning of her stomach. This was followed in a few hours by colicky pains in the right iliac region.

On March 28, she vomited twice. The family, thinking the trouble one of indigestion from the crab salad, gave her a cathartic, and applied antiphlogistine poultices and hot fomentations, with opium. The patient was first seen March 29, at 4 p.m., having a temperature of 102.8°, and pulse of 120. There was great rigidity of the muscles about the right iliac region, with pain most marked below and external to McBurney's point.

The diagnosis of appendicitis was made, and removal to the hospital advised. The family, still thinking that there was a possibility of indigestion, asked for and got a consultant. The diagnosis was confirmed, but although the patient had a fairly easily palpable abdomen, neither the consultant, Dr. Alfred C. Croftan, nor myself could feel the appendix, partly because of the muscular rigidity and partly because of its position. She entered the hospital at 9 p.m., with a temperature of 100.2°; pulse, 108; respirations, 24. She was prepared for operation. On my return to the hospital at 10.30 p.m., the temperature was 100.2°; the pulse had dropped to 88, and there was no pain except on pressure in the right iliac region, and this was less than when first seen. There was, however, greater tympany than when examined at 9 p.m.

I advised operation at once. The family again referred to the crab salad, and said, "It is only a belly ache; see how much better she is." I insisted upon consultation, believing it to be a perilous calm, and it was granted. The consultant agreed with me that operation was imperative, and it was performed at about

1 a.m. The operation took forty minutes, because of many old, firm adhesions of the proximal third of the appendix, and many new of the distal two-thirds. It was a muscle-splitting operation, with skin incision 1⅞ inches long. Later on the day of operation the pulse at 10 a.m. was 88; temperature, 100.2°; respirations, 24. At 6 p.m., pulse, 86; temperature, 98.4°; respirations, 22. During the day she was given liquids by mouth. The day following she was slipped out of bed to use a commode, the pulse and temperature being normal, and two days following was rested in a chair out of bed. The patient left the hospital nine days after operation, following an uneventful recovery.

Specimen from Case II. About four-fifths actual size. Lumen held open showing the two orange seeds.

This one of two recently removed appendices I have brought here as being of sufficient interest to show. This appendix was placed well down on the internal part of the cecum, having a course first anterior, then upwards, then backwards, and downwards, the curved portion being that held by dense old adhesions.

Pathological report in part: Length, 5½ inches. The distal end is greatly enlarged, swollen, and of a dark purplish color, extending two-thirds of the way up the organ to a point where a stricture is found almost obliterating the lumen. The capillaries and vessels over the surface are greatly distended. Upon opening into the lumen there were found two full-sized orange seeds. No free pus, but some fecal contents. The pathological histology of sections of this appendix shows extensive round cell infiltration throughout the mucous membrane, and glands almost entirely destroyed, as well as a part of the muscular coat in places. The bloodvessels were dilated and congested. There was also an interstitial hyperplasia, showing that a chronic form of disease had existed prior to the last attack.

The picture shows an acute catarrhal condition, with extensive necrosis.

Case III.—Mrs. G. W. S., 22 years. First seen August 6, 1904, at noon. Temperature 103° ; pulse, 124 ; respirations, 26. She had marked pain in right iliac region, with rigidity of the muscles on both sides, less in left iliac region. She gave a history of difficult and painful micturition ten days before, with much swelling of the labia, which had subsided under the use of hot douches. Menstruation was normal. There had been no sickness since childhood, but she had not felt well since leaving Arizona in April. The day before, and at 2 a.m., on the day of visit, she had vomited three times, which was attributed to some medicine which she had taken. Vaginal examination showed some discomfort in vagina and tenderness of uterus and adnexa. A diagnosis of appendicitis and infection of uterus and tubes was made, and patient was sent to hospital.

Temperature at entrance at 3 p.m., 103.2 ; pulse, 120 ; respirations, 26. She was prepared for operation for appendicitis. Temperature at 8 p.m., 101° ; pulse, 110 ; respirations, 24. Temperature at 11 p. m., 100° ; pulse, 90 ; respirations, 24.

At this time the patient felt much better, having a less rapid pulse and much lower temperature, but examination revealed increased rigidity of the muscles in the right iliac region, and a particularly painful point below and external to McBurney's point. Vaginal smears showed gonococci. She was operated upon about midnight, and a slightly enlarged congested appendix containing in its distal end one large grape seed was found. The proximal end was somewhat constricted, so that it was barely possible to force through a probe from the distal end. A culture from the lumen showed a pure colon bacillus. An uneventful short recovery followed, she being at the hospital ten days. It is of passing interest to know that she last ate Tokay grapes in Arizona in April ; also that her douche bag had been used by others using a common bathroom.

Case IV.—Mr. A. M., aged 30 ; married. Fairly developed and nourished. Previous history : about one year previous to present attack was sick in bed for three weeks, with a diagnosis of typhoid fever, although no Widal reaction was found.

First seen, October 17, 1902, at about 7 p.m. Facial expression drawn. Movements caused some pain in right inguinal region. He had been in bed two days ; had not vomited, but the pain during the morning of the day seen had been very sharp and colicky. Pulse, 103 ; temperature, 102.8° ; respirations, 24. Pain on palpation of right inguinal region, while marked, allowed sufficient manipulation, so that the appendix could be felt about one and one-half inches outside and below McBurney's point. A diagnosis of appendicitis was made. The patient was sent to the hospital. Passed a good night, sleeping well. In the morning his pulse was 84, and temperature, 99°, but the face was drawn and tympany more marked than on previous night. He was operated on in the morning, and an erect, highly-injected, congested appendix removed. Near the base, the lumen was entirely constricted, and many old adhesions were separated, caused without doubt by the attack of the previous year, then thought to be typhoid. On opening the distended appendix it was found full of a thick, reddish-yellow pus. Cultures showed colon bacillus and staphylococcus.

The night following operation he got out of bed twice during the absence of the nurse to pass urine, and was allowed to get out of bed thereafter. The recovery was uninterrupted.

As briefly as possible, the aim of this paper is to try to reduce the unnecessary mortality due to a hope of recovery without operation. It has been tritely said that so many die of appendicitis because so many get well. Nothing could be more true. The one who gives advice against operation in this treacherous disease must assume a grave responsibility, notwithstanding the patient shows an apparent return to a normal condition, no matter what treatment is used. To say that he has never had a death without operation is only saying that he has been fortunate in not having cases that went on to ulceration, necrosis, perforation, peritonitis and general septicemia. The subsidence of one or more combination of symptoms may not mean recovery, but may mean a far more imminently dangerous condition for the patient. The pulse may return to normal and be of normal volume; the temperature may subside or go below normal. The pain may cease. The dead appendix knows no pain. "After the bowel perforates, all peristalsis rapidly ceases, and the silence of the grave broods over the abdomen." However, the treacherous calm is not a complete one. Something abnormal remains; greater tympany; accelerated pulse; increased pain; drawn facies; or increased muscular rigidity.

As long as the trouble is confined to the appendix, there is no immediate danger. But no one can tell when the trouble will extend to the peritoneum. There are no sharp lines to be drawn, and it is impossible to say when a peritoneum received its infection. Neither is it necessary for the appendix to be perforated for peritonitis and its sequelæ to occur.

These treacherous calms may come at any time during a few hours or days following the acute attack.

G. Dieulafoy well says: "Traitorous calms of appendicitis are often the cause of death. A temporising or hesitating physician notices with eagerness the seeming defervescence of the trouble, wishing to put off or avoid surgical intervention, believing it will always be time to operate later, between attacks, but nevertheless there follow terrible accidents against which surgical treatment is of no avail, and the patient dies."

CONCLUSIONS.

(1) Defervescence of symptoms and apparent better condition of a patient do not always mean recovery, but may be the forerunners of a more dangerous condition.

(2) There being no specific for the disease, no matter what treatment is used, the one who procrastinates should shoulder the responsibility for the death.

(3) When a clear diagnosis is made but one treatment should be advised, that of operation as soon as possible under the conditions, or the golden opportunity may be forever gone.

(4) The physician who does not explain the great dangers of delay and the small comparative danger of operation is doing his patient a serious injustice, which often leads to fatal results.

(5) Operation at the proper time usually greatly shortens convalescence, and eliminates all danger from this cause hereafter.

(6) Procrastination is the greatest cause of surgical deaths, operation often being performed as a last resort, when but little hope of recovery exists.

## Figures and Tables

**Figure f1:**